# Empowering women entrepreneurs: The impact of integrated medical insurance system in China

**DOI:** 10.1371/journal.pone.0337827

**Published:** 2026-02-25

**Authors:** Ling Liu, Xu Zong

**Affiliations:** 1 School of Economics, Peking University, Beijing, China; 2 China National Health Development Research Center, Beijing, China; 3 Helsinki Institute for Demography and Population Health, Faculty of Social Sciences, University of Helsinki, Helsinki, Finland; 4 Max Planck - University of Helsinki Center for Social Inequalities in Population Health, Helsinki, Finland; Alexandru Ioan Cuza University: Universitatea Alexandru Ioan Cuza, ROMANIA

## Abstract

China’s urban-rural Integrated Medical Insurance System (IMIS) has expanded universal health coverage, reducing disparities in healthcare access between urban and rural populations and addressing challenges such as unequal access to medical care and reimbursement barriers for disadvantaged groups. Previous research highlights that health insurance influences medical risk, labor mobility, and income distribution, with women valuing and utilizing health insurance more than men. This study investigates IMIS’s impact on women’s entrepreneurship in China using data from the China Family Panel Studies (CFPS) and the China Statistical Yearbook. A Difference-in-Differences (DID) analysis reveals that IMIS increases the likelihood of women’s entrepreneurship by 9.6 percentage points, with robustness confirmed through multiple tests. The study identifies reduced medical expenditure risk, increased access to high-risk occupations, and enhanced household bargaining power as key mechanisms driving this effect. Heterogeneity analyses show that younger women, those with higher education, rural-urban migrants, women with children, those in poorer health, and those with lower household income get greater benefits, underscoring IMIS’s inclusivity. These findings demonstrate that IMIS promotes women’s entrepreneurship, enhancing economic performance and welfare.

## 1. Introduction

Since China’s reform and opening, economic restructuring and market optimization have expanded self-employment opportunities, leading to a growing number of women pursuing economic independence through entrepreneurship. As a subset of entrepreneurship, self-employment has flourished, with women actively participating despite its limitations in scale and profitability [1,2]. Interest in women self-employment enables a deeper analysis of female entrepreneurship, especially considering the unique health and income risks women face. These challenges, combined with the dual burden of medical insurance and family responsibilities, significantly influence women’s entrepreneurial decisions. Thus, the role of social insurance in stimulating women’s entrepreneurial activities warrants in-depth exploration.

**Fig 1:**
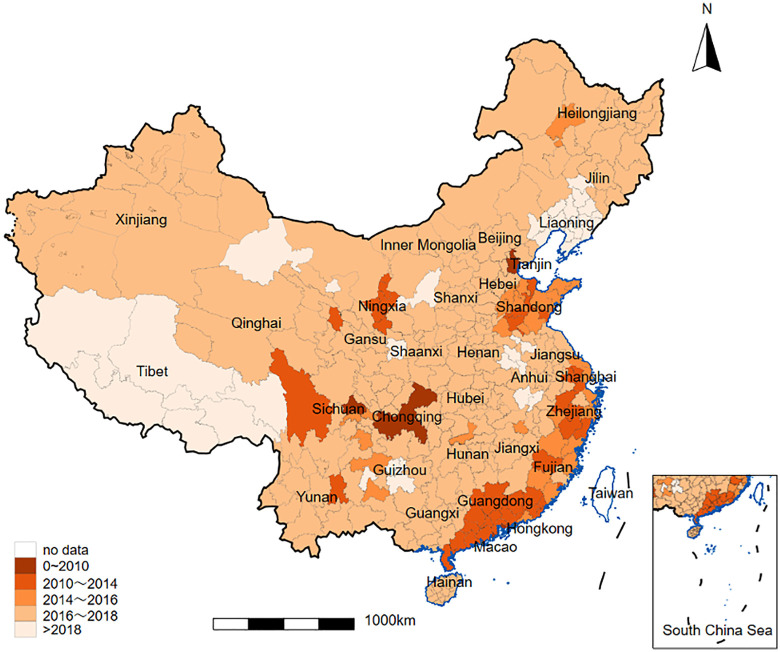
Geographic and temporal variation in the rollout of integrated medical insurance systems. Note: This figure shows the geographic and temporal variation in the rollout of integrated medical insurance systems at the city level across China’s administrative units (provinces, autonomous prefectures, and province-level municipalities). The base map used in this figure is reprinted from the National Administration of Surveying, Mapping and Geo-information’s Standard Map Service (http://bzdt.ch.mnr.gov.cn/) under terms of public domain use, as per the platform’s official guidelines. White cities have no specific information.

The link between health insurance and entrepreneurship stems from China’s health insurance system being tied to the household registration (hukou) system, rendering it a non-portable before reforms. China’s household registration (“hukou”) system, established in the 1950s, records residents’ identities and determines their access to social benefits. By dividing residents into agricultural and non-agricultural (urban) categories, the hukou system shapes individuals’ opportunities in areas such as education, employment, health care, and housing. As shown in Table 1, prior to 2016, China operated three main basic health insurance systems: the Urban Employees’ Basic Medical Insurance (UEBMI), the Urban Residents’ Basic Medical Insurance (URBMI), and the New Rural Cooperative Medical Insurance System (NRCMS). UEBMI provides the highest protection for urban employees in formal sectors, such as government and state-owned enterprises. The NRCMS covers rural residents, primarily for major illnesses, reimbursing hospitalization but not outpatient costs. The URBMI serves urban hukou residents without formal employment, including the unemployed, self-employed, children, and students. In 2016, the State Council released the Opinions on the Integration of the NRCMS and URBMI (GF 2016−3), and to form a residential-based social health insurance program (i.e., URRBMI), guiding local governments to initiate pilot programs to consolidate these medical insurance systems. The Integrated Medical Insurance System (IMIS) has been gradually implemented at the city level across China’s administrative units (provinces, autonomous prefectures, and province-level municipalities), resulting in varying progress in IMIS adoption and medical package offerings among cities within the same administrative units. Fig 1 illustrates the geographical coverage of IMIS in China. Fig 1 was created using a standard map from the official portal of the National Administration of Surveying, Mapping and Geo-information of China, which permits free access, use, and redistribution of standard maps for public purposes.

**Table 1:** Introduction of the three basic health insurance schemes in China (NRCMS, URBMI, and IMIS).

Health insurance type	Pre-IMIS		After IMIS
	NRCMS	URBMI	URRBMI
Target population	Rural residents	Urban non-working residents	Urban (non-working) and rural residents
Implementation year	Launched in 2003 on a national scale	Piloted in 79 cities in 2007; implemented in 2010 on a national scale	Since 2016
Risk pooling unit	County	City district or city (after 2009)	City
Government premium subsidy	Yes	Yes	Yes
Administrative body	NHC	MoHRSS	MoHRSS
Enrollment unit	Household	Household	Household
Reimbursable list for drugs and services	Narrower	Wider	Upgrade to the level of URBMI; Health insurance portability promoted

Note: IMIS refers to Integrated Medical Insurance System; URBMI refers to Urban Resident Basic Medical Insurance; NCMS refers to New Rural Cooperative Medical Scheme; URRBMI refers to Urban and Rural Resident Basic Medical Insurance; MoHRSS stands for Ministry of Human Resources and Social Security; NHC stands for National Health Commission.

The core elements of IMIS are primarily reflected in the following: (1) the city level serves as the basic unit of integration, with provincial-level integration possible where conditions permit; (2) it has eased regional restrictions on enrollment and compensation for medical benefits for rural-urban migrants seeking medical care across regions; (3) the complex and cumbersome application and reimbursement procedures for cross-regional medical care have been simplified, reducing financial burdens and high transportation costs associated with medical service visits. The IMIS reform also aims to improve the rational allocation of healthcare resources in urban and rural areas, enhance the accessibility and quality of healthcare services in marginalized areas, ultimately realizing the vision of health equity for all. Overall, the primary purpose of this policy is to enhance equal access to medical care across provinces and cities and among people of different socio-economic statuses, primarily to meet the basic medical and health needs of those without formal employment and to alleviate the persistent urban-rural medical disparities. Figures released by authorities indicate that IMIS was essentially implemented across all provinces and cities nationwide by 2020, and by the end of 2023, more than two-thirds of the population was covered by IMIS. According to data from the 2023 Statistical Bulletin on Medicare Advancement published by the National Health Insurance Administration (accessed August 23, 2024), the system had achieved extensive national coverage by that time.

IMIS provides a unique context for this research. First, when IMIS was launched, private and other public health insurance (beyond URBMI & NRCMS) where largely unavailable for residents. Moreover, the primary domain where the survey sample collected was in rural areas, where enrollment in private health insurance had barely reached about 5 percent of total population until very recently [3,4]. The lack of a competitive health insurance system allows for the inference that IMIS coverage has a direct effect on women’s health care utilization and economic behavior, with this effect not being confounded by other public or private health insurance [5–7]. Second, the program is universally implemented with high participation rates. Third, the program was gradually implemented across all regions [8,9], which enables the analysis of changes in policy effects over time using robust empirical methodologies. Finally, the most significant impact of IMIS is its facilitation of inter-regional medical service access for China’s large rural-urban migrant population, addressing the initial issue of health insurance non-portability. Therefore, the estimates from this study can also assess the economic benefits of health insurance for women in a state of mobility.

This study employs data from four waves of the China Family Panel Studies (CFPS) and the China Statistical Yearbook and constructs a Difference-in-Differences (DID) model to investigate the causal effects of IMIS on women’s entrepreneurship through a natural experiment. Subsequently, the study constructs a theoretical framework to explore the specific mechanisms through which IMIS influences women’s entrepreneurship. Finally, a subgroup analysis is conducted to examine the heterogeneity in the effects of IMIS on women across subgroups based on health status, parenthood, migration status, and economic conditions. This study provides new empirical evidence on the impact of IMIS on women’s entrepreneurship in the context of improving social equity and pursuing sustainable growth, offering valuable insights for policy design.

Recent studies have explored IMIS’s impact on migrant workers’ entrepreneurship but have not fully examined underlying mechanisms or focused on women specifically [9]. This study fills these gaps, demonstrating that IMIS’s comprehensive benefits stimulate women’s entrepreneurship by reducing medical expenditure risks, enhancing labor mobility, and increasing household bargaining power. These effects are particularly pronounced for younger, better-educated, rural-urban migrant women with children, poorer health, or lower economic status. To our knowledge, this is the first study to identify IMIS as a driver of women’s entrepreneurship.

This study makes three key contributions. First, it enriches health insurance literature by showing that IMIS fosters women’s entrepreneurship, particularly among married and childbearing women. Second, this study analyzes healthcare expenditure risk, labor mobility, and women’s household bargaining power as the primary mechanisms. The results show that IMIS encourages women’s entrepreneurship by lowering catastrophic medical expenditures, increasing women’s participation in high-risk jobs and hours of work, and particularly enhancing women’s relative household incomes and mastery of family affairs. Third, this study responds to calls for more research on women’s entrepreneurship and health insurance systems, with the aim of inspiring further interest in women’s entrepreneurship and encouraging deeper exploration of the institutional influences on women’s business behavior. The economic importance of supporting married and childbearing women’s entrepreneurship has become increasingly evident: motivating married women of childbearing age to start their own businesses not only contributes to economic growth and increased household incomes, but also promotes women’s role within the family and contributes to women’s economic welfare.

The following sections are organized by: Section 2 reviews relevant literatures and formulates the hypotheses; Section 3 provides the data, variables, and model specifications; Section 4 presents the results; and Section 5 and Section 6 contain the discussion and conclusion.

## 2. Literature review and theoretical hypotheses

There are two primary perspectives on defining entrepreneurship: behavioral and occupational [10]. The behavioral perspective emphasizes the entrepreneur’s innovative spirit in pursuing potentially risky opportunities [11]. In contrast, the occupational perspective views entrepreneurs as individuals who work for themselves [10], emphasizing the distinction between waged labor and self-employment. This study adopts the occupational perspective’s definition of entrepreneurship, defining an entrepreneur as an individual whose primary occupation is self-employment or business ownership, including both self-employed individuals with no employees (self-employed entrepreneurs) and business owners who hire others (corporate entrepreneurs). This definition has been widely adopted in Chinese entrepreneurship research [9,12,13].

Previous studies on the effects of health insurance on entrepreneurship among the working-age population have yielded mixed evidence. Some studies show that non-employment-based health insurance promotes entrepreneurship [12,14–17], while others indicate that employer-based health insurance deters it [18–20]. Additionally, universal health insurance has found to reduce self-employment while increase the likelihood of business hiring employees [21].

Potential explanations for the impact of health insurance on entrepreneurship include its positive effects on healthcare utilization and health outcomes [22–24], as demonstrated by Finkelstein et al. (2012) in their landmark Oregon Medicaid experiment showing expanded coverage significantly increased preventive care use [25]. Health insurance also provides risk-sharing for entrepreneurs [12,18], reducing income volatility from health shocks [26], and offers financial risk protection for households [22,27,28], particularly against catastrophic expenditures that disproportionately affect low-income families [29]. Additionally, health insurance reduces the need for precautionary savings against future health risks, as evidenced by Gruber and Yelowitz (1999), who found Medicaid enrollment decreased precautionary savings among eligible households [30]. This influences the recirculation of disposable savings into productive investments [31], such as business startups [32]. Finally, health insurance unlocks the “job lock” phenomenon – where workers retain employment solely to maintain coverage – thereby promoting labor mobility [5,18,21,33–35]. Boyle and Lahey (2010) empirically show that near-retirement employees with employer-based insurance are less likely to transition to self-employment than those with alternative coverage, highlighting how IMIS-like portability could liberate entrepreneurial transitions [36].

While there is a growing body of literature examining the impact of health insurance on individual entrepreneurship, most studies primarily analyze the causal effects of work-bound health insurance on the labor force in general or specific age groups, rarely considering distinct outcomes for women. Within this limited body of literature focused on women’s entrepreneurship, the primary interest lies in the impact of health insurance on women’s entrepreneurship in the U.S., with no clear consensus emerging from the research. Some studies argue that the expansion of medical insurance benefits could stimulate women’s entrepreneurship [6,35]. And some have found no evidence that health insurance is conducive to women’s entrepreneurship [37,38].

Entrepreneurial economics views entrepreneurship as an individual’s career choice based on comparing the expected returns to being an entrepreneur or a wage earner [39,40]. Within this framework, individuals engage in self-employment if their expected utility from self-employment is higher, otherwise they engage in employed work. Individuals’ choices depend on the factors that influence the utility of both occupations.

Building on the existing definitions of entrepreneurship, it is essential to consider how IMIS influences women’s entrepreneurial choices.

Crucially, IMIS addresses the fragmentation of China’s pre-reform health insurance system, which consisted of different urban employees (UEBMI), urban residents (URBMI), and rural schemes (NRCMS) with varying coverage and portability restrictions [41]. Prior research has highlighted how this fragmentation disproportionately hinders women’s labor mobility, rural migrant women working in urban areas face barriers to healthcare reimbursement when returning to their hometowns, effectively “locking” them into formal employment to retain urban coverage [42]. IMIS overcomes these systemic constraints by harmonizing reimbursement rates and enabling claims processing across regions [43], thereby reshaping the risk-reward calculus of entrepreneurship.

Women face two career choices: remaining a wage earner or engaging in entrepreneurship. On one hand, research shows that women face more barriers to entrepreneurship than men [44], including systemic gender biases in access to capital and networks [45]. For example, women’s entrepreneurial activities are generally concentrated in retail and service industries with relatively low profit margins, and they tend to have less access to financing due to discriminatory lending practices [46] and lower risk-sharing capabilities [47]. Risk factors such as illness, which are not conducive to entrepreneurship, pose greater threats to women entrepreneurs [35], particularly in contexts where health insurance coverage is fragmented or inadequate. Entrepreneurship depends on an individual’s financial constraints and entrepreneurial capabilities [48]. Even women with entrepreneurial abilities are more likely to remain wage earners rather than become entrepreneurs due to financial constraints [49], as liquidity shortages disproportionately hinder women’s entry into self-employment [50]. On the other hand, women with working capacity tend to value health insurance more than men [51,52], and women’s utilization of preventive health services is higher and women are more likely than men to be diagnosed with certain conditions [37]. After the implementation of IMIS, women can address these issues more effectively across regions (provinces/cities). Moreover, through IMIS, individuals without formal employment can directly purchase higher-benefit health insurance, significantly reducing the cost for women of leaving full-time employment. Consequently, the impact on women’s job mobility and their choice of entrepreneurship may be more significant due to IMIS’s financial risk protection and provision of higher health benefits. Therefore, this study develops **Hypothesis 1**: IMIS stimulates women’s entrepreneurship.

Additionally, IMIS reduces the risk of catastrophic medical expenditures, a key barrier to entrepreneurship, particularly for women [35]. This financial protection encourages women to pursue entrepreneurial ventures, leading to **Hypothesis 2:** IMIS stimulates women’s entrepreneurship by reducing the risk of catastrophic medical expenditures.

In addition to its direct impact on women’s health expenditures, health insurance may also influence entrepreneurship through indirect channels. IMIS may influence women’s labor mobility. Various employment options may allow women to gain relevant experience or motivate them to pursue different career paths, thereby fostering future entrepreneurship. The portability feature of IMIS enhances women’s job mobility, enabling them to engage in high-risk occupations such as self-employment. As IMIS develops, it could increase the number of individuals engaging in high-risk jobs and acquiring relevant entrepreneurial experience [35]. IMIS could lower the threshold for entrepreneurial competence, thereby contributing to entrepreneurship, particularly among women who face economic and health disadvantages. IMIS reconstitutes labor mobility dynamics by decoupling health security from formal employment. Drawing on job lock theory, women in rigid formal jobs—particularly those with caregiving duties or chronic health needs—often forgo entrepreneurial opportunities to retain employer-sponsored insurance. IMIS disrupts this trade-off, enabling transitions to self-employment by providing portable, income-agnostic coverage. Concurrently, prospect theory suggests that IMIS lowers the perceived risk of entrepreneurship, as out-of-pocket health costs are mitigated. Therefore, this study proposes **Hypothesis 3:** IMIS stimulates entrepreneurship by increasing women’s job mobility. Prior to the introduction of IMIS, women were more engaged in household chores than men due to the division of labor and joint economic decision-making within the household [2]. The lower value of women’s labor may reduce their wage levels, thereby negatively affecting their bargaining power and decision-making authority within the household. This creates an imbalance between women and their husbands in terms of wages and bargaining power. Consequently, when women start a business, they have fewer opportunities to benefit from family financial support. On the other hand, IMIS may incentivize women to become more active in high-risk jobs. Hence, women’s wage incomes may increase, particularly for those who were previously unemployed or had lower salaries, and their bargaining power within the household (i.e., relative salary ratios between women and their husbands) may also improve. In essence, IMIS enhances women’s intra-household bargaining power by raising their relative income, which in turn boosts their participation in intra-household knowledge and capital transfers, thereby stimulating women’s entrepreneurship [53]. Therefore, this study formulates **Hypothesis 4:** IMIS empowers women’s entrepreneurship by increasing their relative household incomes and authority in family matters.

## 3. Research design

### 3.1. Data and sample selection

This study utilizes two main sources of datasets. First, the individual- and household-level data are derived from the China Family Panel Studies (CFPS), initiated in 2010 by the Institute of Social Science at Peking University. CFPS regularly obtains ethical approval from the Biomedical Ethics Committee of Peking University before carrying out the corresponding data collection work. The CFPS data encompass a representative spectrum of the population, covering more than 14,000 households and their entire household members in the 25 provinces, municipalities, and autonomous regions of China (94.5% of the mainland’s population). These data include detailed information on demographic characteristics, work status, wage income, and family relationships. For this study, the sample from 2014 to 2020 was specifically selected to include only participants who are married and have spouse-related information available. Detailed information on individuals’ work, medical expenditures, and intra-household empowerment was only started in 2014, making earlier data unsuitable for this analysis. Given the study’s sample requirements, participants must meet eligibility criteria, including being able to work and being married, specifically targeting married women aged 18–60. Additionally, the sample was screened to include only those who participated in the survey within the respective years and could clearly indicate their work status. Ultimately, only participants who completed at least one wave of the survey and had no missing core variables or illogical responses were retained, resulting in a final sample of 19,755 women.

There are two reasons for selecting married women as the sample for this study. From an empirical standpoint, consistent with existing studies, married women are more engaged in self-employment than unmarried women [54]. Furthermore, within this group, young children significantly influence the rate of self-employment, making them a relevant demographic for this study [2]. Single women encounter different constraints in making employment decisions compared to married women and are thus not the focus of this study.

The CFPS meets our research needs in several ways. First, it features a panel structure designed to track each respondent regardless of their residential location. Second, the dataset records precise job information, crucial for analyzing the dynamic effects of labor supply. Third, the data includes detailed information on households’ medical, economic, and internal dynamics, facilitating a deeper exploration of the underlying mechanisms. Additionally, province-level variables are compiled from the annual China Statistical Yearbook. Economy-related variables use the 2014 CPI (consumer price index) as a basis to enable year-over-year comparisons.

### 3.2. Variable definition and modeling

Dependent variable: Women’s entrepreneurship, defined as a dummy variable based on the primary occupation reported by women, assigned a value of 1 if the response is “private enterprise/self-employed/other self-employed” and 0 otherwise.

Independent variable: IMIS. The study examines the impact of IMIS from 2016 onward, excluding regions that implemented it prior to 2016. The specific year of IMIS implementation for each city is sourced from detailed program implementation documents for each region. The IMIS dummy variable is set to 1 if the interview occurred after the region introduced IMIS, and 0 otherwise.

Control variables: Considering that individual, household, and province-specific confounders all impose effects on women’s entrepreneurship in various degrees, this study corrects for all three dimensions [55]. The specific definitions of these variables are provided in Table A1 in S1 Appendix. Specifically, demographic characteristics include age, age squared, years of education, household register status, party affiliation, health status, health insurance coverage, and internet use. All variables are dummy variables except for age, which is treated as a continuous variable. According to previous literature, the age squared variable is designed to capture the potential non-linear effects of age on women’s entrepreneurship [9,13]. Household characteristic variables include child and elderly dependency ratios, family size, annual household consumer expenditures, annual per capita household assets, and clan culture, all of which significantly influence the pattern of resource allocation within the household. Province-level control variables include population density (persons per square kilometer), regional GDP per capita, unemployment rate and CPI, which controls for the economic status of the region in which the woman resides. The number of hospitals and doctors per 10,000 residents is also included, controlling healthcare conditions in the province. These control variables, as demonstrated in previous studies, significantly impact the findings and allow for a more accurate estimation of IMIS’s effect on women’s entrepreneurship.

The descriptive statistics of the variables from the final samples are presented in Table 2.

**Table 2:** Summary statistics.

Variables	Total Sample	Treatment Sample	Control Sample	Difference
Observation	19,755	5,944	13,811	
**Panel A: Outcome variables**				
Women’s Entrepreneurship	0.285	0.560	0.167	0.394^***^
	(0.452)	(0.496)	(0.373)	
**Panel B: Individual variables**				
age	43.150	44.161	42.715	1.446^***^
	(10.219)	(10.081)	(10.247)	
age2	19.663	20.518	19.296	1.222^***^
	(8.540)	(8.607)	(8.485)	
education	6.870	7.211	6.723	0.452^***^
	(4.204)	(4.121)	(4.231)	
hukou	0.155	0.143	0.161	−0.017^***^
	(0.362)	(0.350)	(0.367)	
communist	0.023	0.021	0.024	−0.003
	(0.150)	(0.143)	(0.153)	
medsure_dum	0.914	0.927	0.909	0.018^***^
	(0.280)	(0.260)	(0.288)	
health	0.674	0.692	0.666	0.026^***^
	(0.469)	(0.462)	(0.472)	
internet	0.424	0.579	0.358	0.221^***^
	(0.494)	(0.494)	(0.479)	
**Panel C: Household variables**				
elder_p	0.111	0.128	0.104	0.024^***^
	(0.254)	(0.282)	(0.240)	
child_p	0.345	0.403	0.320	0.083^***^
	(0.390)	(0.455)	(0.356)	
familysize	4.614	4.738	4.561	0.177^***^
	(1.829)	(1.881)	(1.803)	
hhexp (10 thousand yuan)	6.661	7.339	6.369	0.969^***^
	(6.281)	(6.610)	(6.111)	
hhpca (10 thousand yuan)	11.194	14.133	9.929	4.203^***^
	(19.578)	(22.900)	(17.813)	
clan	0.213	0.2263	0.208	0.018
	(0.982)	(0.969)	(0.988)	
**Panel D: Province level variables**				
gdp_pc	5.087	5.742	4.805	0.937^***^
	(3.708)	(4.542)	(3.245)	
PD	0.041	0.047	0.038	0.008^***^
	(0.045)	(0.048)	(0.043)	
perhos	0.518	0.257	0.631	−0.374^***^
	(0.394)	(0.067)	(0.422)	
perdocs	23.746	26.521	22.552	3.969^***^
	(7.200)	(7.500)	(6.724)	
unemp	3.251	3.237	3.258	−0.021^**^
	(0.577)	(0.294)	(0.663)	
cpi	101.983	102.263	101.863	0.400^***^
	(0.448)	(0.419)	(0.404)	

Notes: Standard errors are in parentheses. ^***^, ^**^ and ^*^ denote t-test significance levels for differences in means between the urban and rural health insurance integration subsamples. Standard errors are clustered at the individual level. ^***^, ^**^, and ^*^ denote significance levels of 1%, 5%, and 10%, respectively. Control variable definitions: age = years of age; age2 = age squared; education = years of education; hukou = household registration status; communist = party affiliation; health = health status; medsure_dum = health insurance coverage; internet = internet use; elder_p = elderly dependency ratio; child_p = child dependency ratio; familysize = family size; hhexp (10,000 yuan) = annual household consumer expenditures; hhpca (10,000 yuan) = annual per capita household assets; clan = clan culture; gdp_pc = regional GDP per capita; PD = population density (persons per square kilometer); perdocs = number of hospitals and doctors per 10,000 residents; unemp = unemployment rate; CPI = consumer price index.

Table 2 presents the means and standard deviations of all variables for the full sample, as well as for the subsamples of the treatment and control groups, and includes tests of variability in variable means for the subsamples in the last column. The results show that 28.5% of working-age women are engaged in entrepreneurship. In addition, the sample population in the treatment group is better off than the control group in terms of education level, health status, family economic status and urban economic and medical development.

### 3.3. Research methodology

This study focuses on areas where integration occurred after 2016, with regions that implemented IMIS before the interview year serving as the treatment group. A staggered DID approach is employed to analyze the impact of IMIS on women’s entrepreneurship. The model used in this study is specified as follows:


$$Y_{ijt}=\alpha_{\text{0}}+\alpha_{\text{1}}IMIS_{it}+\beta X+\lambda_{j}+\delta_{t}+\varepsilon_{ijt}$$ (1)

i represents the woman chosen for interview, j  denotes the region where women live, and t represents the year. $Y_{ijt}\;$ indicates the dependent variable, namely the dummy of women’s participation in entrepreneur; IMIS  represents independent variable to indicate whether IMIS covers the region; X represents the control variable influencing women’s entrepreneur; $\lambda_{i}$ represents the dummy variable of the province, which is used to control the province effect; $\delta_{t}$ represents the year dummy variable, which is used to control the time effect; $\varepsilon_{ijt}$ is error item.

## 4. Results

### 4.1. Baseline model

Table 3 presents the effect of IMIS on women’s entrepreneurship. The results indicate that the positive effect of IMIS on women’s entrepreneurship remains statistically significant across regression models, even as demographic, household-level, and province-level variables are progressively added. Specifically, the baseline model in column (4) show that IMIS significantly increases the likelihood of women’s entrepreneurship by 9.6 percentage points, supporting Hypothesis 1. This finding is particularly meaningful for China as it continues to deepen health care reforms aimed at ensuring the well-being of women.

**Table 3:** Baseline model: Estimation of the effect of IMIS on women’s entrepreneurship.

Variables	Women’s Entrepreneurship			
	(1)	(2)	(3)	(4)
IMIS	0.042^***^	0.043^***^	0.040^***^	0.096^***^
	(0.014)	(0.014)	(0.013)	(0.015)
age		0.005^*^	0.004	0.004
		(0.003)	(0.003)	(0.003)
age2		−0.005	−0.004	−0.004
		(0.003)	(0.003)	(0.003)
education		0.004^***^	0.003^***^	0.003^***^
		(0.001)	(0.001)	(0.001)
hukou		−0.059^***^	−0.074^***^	−0.075^***^
		(0.012)	(0.012)	(0.012)
communist		−0.003	−0.007	−0.010
		(0.023)	(0.023)	(0.023)
medsure_dum		0.011	0.008	0.007
		(0.011)	(0.011)	(0.011)
health		0.028^***^	0.022^***^	0.020^***^
		(0.007)	(0.007)	(0.007)
internet		0.041^***^	0.030^***^	0.027^***^
		(0.008)	(0.008)	(0.008)
elder_p			0.008	0.009
			(0.013)	(0.013)
child_p			−0.014	−0.015
			(0.010)	(0.010)
familysize			0.009^***^	0.009^***^
			(0.002)	(0.002)
Log (hhexp)			0.023^***^	0.022^***^
			(0.004)	(0.004)
Log (hhpca)			0.034^***^	0.034^***^
			(0.004)	(0.004)
Log (clan)			−0.022^*^	−0.023^*^
			(0.012)	(0.012)
Log (gdp_cp)				−0.130^***^
				(0.049)
Log (PD)				28.649^***^
				(5.754)
perhos				−0.096^***^
				(0.015)
perdocs				−0.007^***^
				(0.002)
unemp				0.066^***^
				(0.013)
cpi				0.096^***^
				(0.010)
Observations	19,754	19,754	19,754	19,754
Adjusted R-squared	0.250	0.255	0.264	0.271

Notes: This table reports the effect of the IMIS (Integrated Medical Insurance System) on participation in entrepreneur of women in China. The regression model is specified in Equation (1). Dependent variable is the women’s entrepreneurship dummy. Column (1) contains no control variables. Column (2) introduces individual-level control variables, while column (3) adds household-level control variables based on column (2). Column (4) further includes province-level control variables, year fixed effects and province fixed effects. All the variables are defined in Table A1 in S1 Appendix. Robust standard errors clustered at the individual level are reported in parentheses. ^***^ < 0.01, ^**^ < 0.05, ^*^ < 0.1. Control variable definitions: age = years of age; age2 = age squared; education = years of education; hukou = household registration status; communist = party affiliation; health = health status; medsure_dum = health insurance coverage; internet = internet use; elder_p = elderly dependency ratio; child_p = child dependency ratio; familysize = family size; Log(hhexp) = logarithm of household consumer expenditures; Log(hhpca) = logarithm of per capita household assets; Log(clan) = logarithm of clan culture index; Log(gdp_pc) = logarithm of regional GDP per capita; Log(PD) = logarithm of population density; perdocs = number of hospitals and doctors per 10,000 residents; unemp = unemployment rate; CPI = consumer price index.

The influence of control variables on women’s entrepreneurship is further explored. Column (2) reveals that education, health, family size, internet use, household consumer expenditures, and household assets all significantly enhance women’s entrepreneurship, consistent with the findings of previous studies. Specifically, whether women become entrepreneurs is closely related to individual backgrounds that correlate with higher human capital, which, as previous entrepreneurship research has shown, often provides a solid foundation for motivating individuals to start their own businesses. The higher a woman’s level of education, the greater her learning ability and knowledge base, and the higher the likelihood of entrepreneurship. Financial literacy acquired through education increases women’s economic and social resources, thereby encouraging entrepreneurial ventures. Similarly, health, family size, internet usage, household consumer expenditures, and assets contribute to human capital, widely recognized as a key pathway to entrepreneurship [56].

### 4.2. Robustness tests

#### Parallel trend test.

The event study method is employed to verify that the treatment and control groups share the same time trend prior to the policy shock. The specific model is formulated as follows:


$$\begin{array}{c} Y_{ijt}=\alpha_{\text{0}}+\alpha_{\text{1}}Pre_{it}^{\text{2}}+\alpha_{\text{2}}Pre_{it}^{\text{3}}+\alpha_{\text{3}}Pre_{it}^{\text{4}}+\alpha_{\text{4}}Pre_{it}^{\text{5}}+\alpha_{\text{5}}Post_{it}^{\text{0}}\\ \;\;\;+\;\alpha_{\text{6}}Post_{it}^{\text{1}}+\alpha_{\text{7}}Post_{it}^{\text{2}}+\alpha_{\text{8}}Post_{it}^{\text{3}}+\beta X_{ijt}+\lambda_{j}+\delta_{t}+\varepsilon_{ijt} \end{array}$$ (2)

Where $Pre_{it}^{s}\;(\text{s}=\text{2},\;\text{3},\;\text{4},\;\text{5})$ is a dummy variable that equals 1 if woman *i* ’s region *j* is before the IMIS rollout in year t+s and 0 otherwise. $Post_{it}^{k}\;(\text{k}=\text{0},\;\text{1},\;\text{2},\;\text{3})$ is a dummy variable that equals 1 if region *j* is after the IMIS rollout in year t−k and 0 otherwise. The year when IMIS is initially carried out in a given region is defined as the base year. Consistent with Equation (1), all other variables remain the same. If the parallel trend hypothesis is robust, none of the coefficients for $Pre_{it}^{s}$ (s=2, 3, 4, 5) is supposed to be significant.

**Fig 2:**
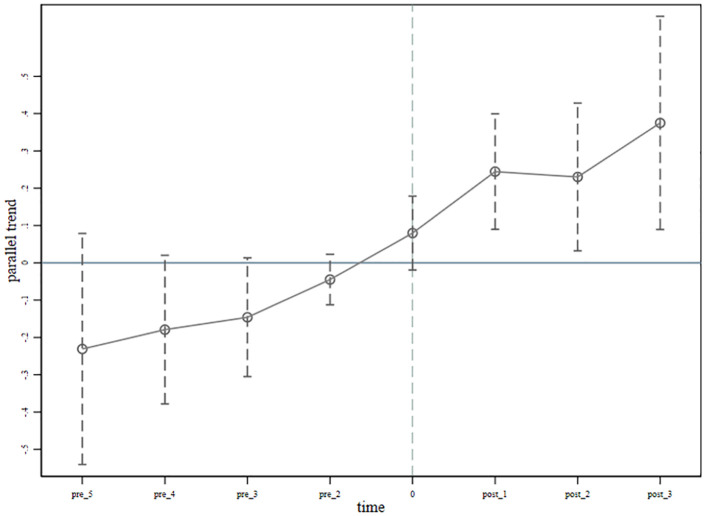
The dynamic effect of IMIS reform on women’s entrepreneurs (TWFE). Note: IMIS refers to Integrated Medical Insurance System. The results from estimating according to the Equation (2) event study method. Control variables are included at individual and household levels as shown in Table 2. Standard errors are clustered at the individual level.

The dynamic effects of IMIS on women’s entrepreneurship are depicted in Fig 2. The figure illustrates that parallel trends were established before the implementation of IMIS. The positive treatment effect observed after the base year of IMIS further validates the robustness of the baseline results. However, the impact of IMIS on women’s entrepreneurship is not significant in the base year. A potential explanation is that IMIS mainly benefits health protection in the short period, which gradually influences women’s entrepreneurial behavior through other mechanisms (e.g., improved health drives increased work capacity, etc.).

#### Heterogeneity of treatment effects.

Table 4 presents the estimated coefficients and 95% confidence intervals (CIs) for IMIS, using the dummy variable for women’s entrepreneurship as the dependent variable. Estimated coefficients are provided for two versions of a two-way fixed effects (TWFE) model: a simplified model without covariates, including only individual and year fixed effects, and a full model with all covariates and fixed effects (Equation (2)). Three additional event study estimates are conducted using recently developed robust DID estimators: the *DIDm* estimator proposed by De Chaisemartin and d’Haultfoeuille (2020), are also conducted, the *csdid* estimation method proposed by Callaway &Sant’Anna (2021), and the estimation method *eventstudyinteract* proposed by Sun & Abraham (2021) [57–59]. In addition to the above tests, estimates with reference to Wooldridge (2021), and Gardner’s (2021) are also conducted [60,61]. The results show consistency with the benchmark results, verifying that the estimation results in this paper are robust and reliable.

**Table 4:** Robust DID estimations.

	Coefficient	SE	Lower 95% CI	Upper 95% CI
	(1)	(2)	(3)	(4)
TWFE (full sample, no controls)	0.230	0.092	0.038	0.422
TWFE (full sample, full controls)	0.080	0.039	0.004	0.155
(De Chaisemartin and d’Haultfoeuille, 2020)	0.039	0.017	0.005	0.073
(Callaway and Sant’Anna, 2021)	0.082	0.020	0.044	0.121
(Sun and Abraham, 2021)	0.070	0.019	0.032	0.108
(Wooldridge, 2021)	0.086	0.015	0.056	0.115
(Goodman-Bacon, 2021)	0.049	0.018	0.014	0.084
(Gardner, 2021)	0.110	0.024	0.064	0.157

Notes: This table reports estimated effect of IMIS (Integrated Medical Insurance System) on participation in entrepreneur of women based on various regression specification. (1) a parsimonious TWFE estimator with no controls is adopted in the full sample, (2) the baseline TWFE estimator with the full list of controls and time trends in the full sample, (3) the *DIDm* estimator of De Chaisemartin and d’Haultfoeuille (2020), (4) the *csdid* estimator of Callaway and Sant’Anna (2021), (5) estimation with eventstudyinteract of Sun and Abraham (2021), and (6) the *jwdid* estimator of Wooldridge (2021), (7) the estimation of Goodman-Bacon (2021), and (8) the estimation of Gardner (2021). The dependent variable is a participation dummy. Standard errors are all clustered at individual level. *csdid* coefficients are calculated by the doubly robust IMIS estimator based on stabilized inverse probability weighting and ordinary least squares (the *dripw* command in Stata).

Further, DID diagnostic tests are performed. First, the Bacon decomposition method proposed by Goodman-Bacon (2021) is utilized [62], and the analysis is conducted using a balanced panel. This method suggests that if there are heterogeneous treatment effects in DID, the estimation may be biased due to the negative weighting problem of the two-way fixed-effects estimator, even if the parallel-trend assumption holds. Accordingly, Table A2 in S1 Appendix presents the specific results of the Bacon decomposition. Forbidden comparisons (Late vs. Early) produce DID estimates that are close to zero.

#### Placebo test.

**Fig 3:**
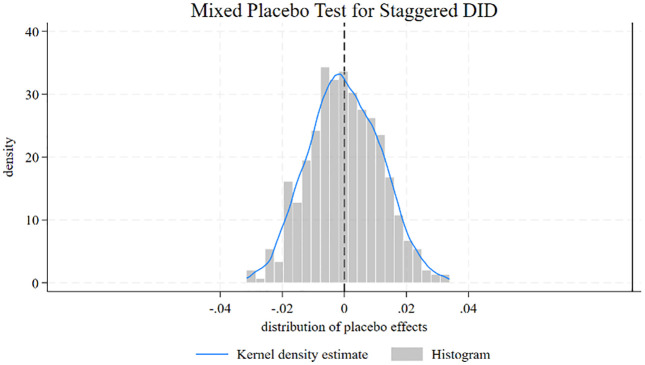
Placebo test. Note: Mixed placebo test for IMIS (Integrated Medical Insurance System) using both pseudo-treated individuals and pseudo-treatment times is employed using the Stata command didplacebo  developed by Chen et al. (2023). It uses both pseudo-treated groups and pseudo-treatment year. Specifically, the pseudo-treatment year for each city is randomly selected from this interval based on the earliest versus latest year of IMIS in the sample, conducted the TWFE estimation, and reproduced 500 times to give the distribution of the placebo effect. The specification of TWFE estimation follows Equation (1).

A mixed placebo test for DID is conducted using the command didplacebo, developed by Chen et al. (2023) [63]. The results indicate that the average treatment effect is significant at the 5% level. Detailed information is provided in Table A3 in S1 Appendix. Fig 3 demonstrates that the baseline results are also robust.

#### Other robustness tests.

**Alternative variables.** First, the definition of entrepreneurship is expanded to include entrepreneurial forms that involve hiring employees, based on CFPS data. Second, self-reported IMIS coverage by women is used as a proxy independent variable. The estimation results for the alternative variables are presented in columns (1) and (2) of Table 5, and the estimated coefficients are all significantly positive, indicating that the conclusions obtained from the baseline model remain valid.

**Table 5:** Additional robustness tests.

	Alternative Variables		PSM-DID			Other robustness	
	Business	self-reported IMIS	NN	Kernel	Radius	MEMI	Endogeneity
	(1)	(2)	(3)	(4)	(5)	(6)	(7)
IMIS	0.019^**^	0.032^**^	0.057^***^	0.056^***^	0.057^***^	0.095^***^	0.263^**^
	(0.009)	(0.014)	(0.018)	(0.018)	(0.018)	(0.015)	(0.103)
N	19,746	19,754	10,468	10,453	10,468	19,755	10,473
Adj. R^2^	0.105	0.269	0.184	0.185	0.184	0.364	0.393

Notes: IMIS refers to Integrated Medical Insurance System. Columns (1)-(2) report the results obtained by replacing the dependent variable with yes/no to starting a business and the independent variable with women’s self-reported IMIS coverage, respectively, and then estimating according to Equation (1). Columns (3)-(5) report results from PSM-IMIS regressions based on 1:1 nearest neighbor matching, kernel matching, and radius caliper matching. Column (6) reports the results of the estimation of Equation (1) with the inclusion of “Mass Entrepreneurship and Mass Innovation” (MEMI). Column (7) presents the results of estimating Equation (1) for a subsample of provinces that launched IMIS in 2017. Control variables and fixed effects are the same as shown in Table 3. Standard errors are clustered at the individual level. ^***^, ^**^, and ^*^ denote significance levels of 1%, 5%, and 10%, respectively.

**Propensity score matching difference in difference estimation (PSM-DID).** To avoid sample selection bias, PSM-DID is also employed for robustness testing. The matching methods are 1:1 nearest neighbor, kernel, and radius caliper methods, after which the DID estimation is obtained using Equation (1). The PSM-DID results are reported in Table 5, columns (3) – (5), and all the results confirm that Hypothesis 1 is robust, as inferred in this study. Figure B1 in S1 Appendix presents the kernel density plots of the treatment and control groups before and after matching for the different PSM methods.

**Exclusion of other confounding policies.** In addition to the IMIS shock, the State Council issued the “Opinions on Several Policy Measures on Strongly Promoting Mass Entrepreneurship and Mass Innovation” (GF 2015−32) (hereinafter, MEI) during the survey collection period. This policy reform may also have impacted women’s entrepreneurship. In the subsequent year, the central government introduced various policy documents to foster individual entrepreneurship and innovation, marking the period of interest for this study. To control for the effect of MEI, a dummy variable representing the year of MEI is added to Equation (1), with the regression results presented in column (6) of Table 5. As a result, the effect of IMIS on women’s entrepreneurship remains robust.

**Endogeneity considerations.** Due to the progressive nature of the IMIS, regions that implemented IMIS in 2017 (53% of the sample) are more likely to be affected by exogenous durations imposed by national policy than those that carried out integration processes closer to 2017 [9]. Hence, it would be more helpful to mitigate the endogeneity problem posed by self-referral decision with the estimation of a subsample of provinces that initiated IMIS in 2017. The regression results are presented in column (7) of Table 5, and they remain robust.

### 4.3. Mechanism analysis

The key channels through which IMIS enhances women’s entrepreneurship warrant further investigation. This section considers three possible mechanisms that may explain the link between IMIS and women’s entrepreneurship: healthcare expenditure risk, labor mobility, and household bargaining power. Whereas earlier research emphasized risk transfer from health insurance policies [64], IMIS operates uniquely through occupational mobility and family bargaining power – channels that employers lack in tying UEBMI.

First, IMIS intuitively reduces medical risk [22,24] and provides financial risk protection for households [14,22,65]. To test whether IMIS increases women’s financial risk protection by reducing healthcare risk, catastrophic medical expenditures (cme) are measured as the level of healthcare risk. As reported in column (1) of Table 6, the interaction term IMIS*cme is significantly negative at the 1% significance level. This suggests that IMIS has a greater impact on entrepreneurship in households with higher medical risks. Thus, IMIS influences women’s entrepreneurship by reducing medical risks. IMIS provides financial risk protection and mitigates the entrepreneurial risks that women must bear. These results validate Hypothesis 2.

**Table 6:** Mechanism analysis: Catastrophic medical expenditures, labor mobility, women’s household bargaining power.

Variables	Women’s Entrepreneurship				
	(1)	(2)	(3)	(4)	(5)
IMIS	0.119^***^	0.186^***^	0.214^***^	0.128^***^	0.228^***^
	(0.015)	(0.016)	(0.041)	(0.018)	(0.032)
IMIS*cme	−0.086^***^				
	(0.017)				
catastrophic medical expenditures (cme)	−0.019^***^				
	(0.007)				
IMIS*hrj		−0.218^***^			
		(0.015)			
high-risk work (hrj)		0.180^***^			
		(0.008)			
IMIS*wwh			−0.038^***^		
			(0.010)		
weekly working hours (wwh)			0.032^***^		
			(0.006)		
IMIS*wbs				−0.010^***^	
				(0.004)	
women’s bargain score (wbs)				0.005^***^	
				(0.002)	
IMIS*wri					−0.071^**^
					(0.028)
women’s relative income (wri)					0.046^***^
					(0.014)
Observations	19,754	19,754	11,174	19,754	13,700
Adj. R^2^	0.274	0.295	0.286	0.272	0.231

Notes: IMIS refers to Integrated Medical Insurance System. Column (1) reports the result that the interaction IMIS*cme is included and conducts regression analysis based on Equation (1). catastrophic medical expenditures (cme) are measured as the level of health care risk using a dummy variable equals 1 if health care expenditures account for more than 40 percent of household expenditures and 0 otherwise. Column (2) reports the result that interaction terms for IMIS*hrj is included and conduct regression analyses based on the baseline model. High-risk work (hrj) is defined as self-employment and paid work in the private sector, which is characterized by lower job security stability but more pay than employment in the public sector. A dummy variable equals 1 if the woman’s main type of work is high-risk, and 0 otherwise. Column (3) reports the result that the interaction term IMIS*wwh is constructed and conduct a regression analysis based on Equation (1). Women’s weekly working hours (wwh), a continuous variable is derived from the question “How many hours per week do you typically spend at work?”. Columns (4)-(5) show the results of adding women’s bargain score (wbs), women’s relative income regressions (wri) to Equation (1), respectively. Women’s bargain score is derived from respondents’ responses to five questions in the CFPS questionnaire dealing with the distribution of decisions on important household matters. There are five main questions in the CFPS questionnaire about main household affairs of families: household expenditure distribution, risky financial decisions, house purchase, child discipline and spending on high consumer goods. After identifying husbands and wives in the household, the value of the women’s bargain score is equal to 1 if the household affairs are “decided by the wife”, and 0 otherwise. The five scores are summed to give a total score for women’s household bargain. The relative wage income of women in the household by constructing: woman’s salary income/ (woman’s salary income + spouse’s salary income).

Control variables and fixed effects are the same as shown in Table 3. Standard errors are clustered at the individual level. ^***^, ^**^, and ^*^ denote significance levels of 1%, 5%, and 10%, respectively.

Hypothesis 3 posits that rural and urban health insurance may promote women’s entrepreneurship by increasing their participation in high-risk jobs. It is well established that women generally have a more conservative attitude towards risk compared to men, which affects their entrepreneurial capacity. IMIS, however, alleviates women’s medical concerns, encourages them to engage in high-paying and high-risk occupations, and provides more labor choices, thereby building women’s experience in high-risk jobs, which in turn enhances their entrepreneurial potential [66]. This logic is tested by using high-risk work as a mechanism. The results in column (2) of Table 6 indicate that the interaction term IMIS*hrj is significantly and positively associated with women’s entrepreneurship and has economic significance, suggesting that engaging in high-risk jobs enhances the impact of IMIS on women’s entrepreneurship. These findings suggest that IMIS has a favorable and supportive effect on women’s labor mobility, particularly in terms of entrepreneurship.

Furthermore, this study delves deeper into women’s labor participation by exploring the impact of IMIS on women’s weekly working hours (wwh). In this analysis, the sample is restricted to respondents for whom detailed job information is available at the time of the survey, as such information is required to measure time spent on both work and housework. The results are presented in column (3) of Table 6, showing that IMIS increases the number of hours women work and has a more significant impact on entrepreneurship among women who work fewer hours per week. IMIS also deepens women’s labor force participation, increasing their economic value and providing them with greater entrepreneurial opportunities.

Another possible mechanism is women’s intra-household bargaining power. Intra-household dynamics are an important mediator of women’s entrepreneurship [67,68]. IMIS enhances women’s incomes, and as women’s share of income increases, they exercise greater bargaining power within their household [69]. Increased bargaining power in major household matters reduces inequality in intra-household resource allocation, which in turn enhances women’s enterprise capacities and access to entrepreneurial capital. This study uses two main proxy variables to describe women’s intra-family bargaining power: subjective and objective. The objective variable is women’s relative wage income within the household, defined as a woman’s salary income divided by the sum of her own and her spouse’s salary incomes. Couple identification is based on information from the CFPS Adult Database and the Family Connections Database. Measuring women’s household bargaining power directly in terms of relative wage income may introduce endogeneity problems [70]. However, family bargaining power dynamics can also be inferred from the subjective preferences of the couple, such as who has the final say on vital family matters. Thus, another variable is the women’s total household bargaining score. The higher the score, the greater the woman’s bargaining power within the household.

Columns (4) and (5) of Table 6 present estimates of women’s household bargaining power. The results indicate that IMIS enhances women’s household bargaining power. The coefficient on the interaction variable is significantly negative, suggesting that IMIS has a more pronounced effect on women’s entrepreneurship when household bargaining power is lower. These findings validate Hypothesis 4.

In summary, IMIS could influence financial risk protection, labor mobility, hours of work, and household bargaining power, thereby stimulating women’s entrepreneurship.

### 4.4. Heterogeneity analysis

The total sample is divided into two subsamples based on the median age of 45. As shown in Table 7, Panel A, IMIS increases the likelihood of women’s entrepreneurship by 10.6 percentage points among women under 45. According to life-cycle theory, this is most likely due to the fact that women in the reproductive age group of 18–45 years may need to utilize health care resources frequently, and the support of health insurance policies to cover maternal health care has a direct impact on their health and financial situation.

**Table 7:** Heterogeneity results.

Variables	(1)	(2)	(3)
**Panel A: Subgroups by age**			
	age < 45	age>=45	
IMIS	0.106^***^	0.086^***^	
	(0.022)	(0.020)	
Observations	9,688	10,066	
**Panel B: Subgroups by education**			
	Larger	Less	
IMIS	0.131^***^	0.071^***^	
	(0.026)	(0.018)	
	6,384	13,370	
**Panel C: Subgroups by rural to urban migrants**			
	Migrants	Not migrants	
IMIS	0.141^***^	0.087^***^	
	(0.030)	(0.017)	
	5,105	14,649	
**Panel D: Subgroups by childbirth**			
	Have a child	Have no child	
IMIS	0.104^***^	0.056^**^	
	(0.019)	(0.025)	
	13,207	6,547	
**Panel E: Subgroups by health status**			
	Better	Average	Worse
IMIS	0.083	0.085^***^	0.122^***^
	(0.054)	(0.023)	(0.030)
	1,298	8,072	4,446
**Panel F: Subgroups by economic status**			
	Lowest 1/3	Middle 1/3	Highest 1/3
IMIS	0.108^***^	0.083^***^	0.088^***^
	(0.027)	(0.026)	(0.026)
	6,579	6,584	6,575

Notes: IMIS refers to Integrated Medical Insurance System. Panel A is an analysis of two subsamples divided according to median age 45. Panel B is an analysis of subsamples grouped according to mean years of education, with poorer and better education. Panel C is an analysis of subsamples by migrant or non-migrant. Panel D is based on an analysis of two subsamples of women with or without children. Panel E provides three subgroup analyses based on women’s self-reported health status of worse, fair, and better. Panel F is a sub-sample analysis based on women’s household consumer expenditures divided into tertiles. Control variables and fixed effects are the same as shown in Table 3. Standard errors are clustered at the individual level. ^***^, ^**^, and ^*^ denote significance levels of 1%, 5%, and 10%, respectively.

The samples with lower and higher levels of education are then analyzed separately. The results of Panel B show that IMIS is more significant in increasing entrepreneurship among better educated women, suggesting that educational resources are closely linked to women’s entrepreneurship and skill-driven opportunity switching, which is consistent with findings from previous studies [71].

Next, a subsample analysis based on migration status is conducted, with results reported in Panel C of Table 7. The results indicate that IMIS is more conducive to entrepreneurship among rural-urban migrant women workers. Migrants may be enriched by exposure to urban markets (a human capital asset). This difference suggests that the role of IMIS is more pronounced among socio-economically disadvantaged women. The stronger effect on migrants reflects both reduced portability barriers (mobility mechanism) and their heightened exposure to uninsured medical risks pre-IMIS.

Subsequently, based on an analysis of two subsamples of women with and without children, Panel D presents results showing that IMIS significantly promotes entrepreneurship among women with children. This may be explained by the entrepreneurial choices made by married women with children in balancing family responsibilities with work, due to the fact that entrepreneurship is a better way of managing working hours than other types of work [2,54,72].

Three subgroup analyses based on changes in women’s self-reported health status are conducted, and Panel E presents the results, indicating that the effect of IMIS on entrepreneurship is more significant for women with poorer health, suggesting the welfare effect of health insurance on disadvantaged groups.

Finally, Panel F presents a subsample analysis based on economic level tertiles. The results suggest that the incentivizing effect of IMIS on entrepreneurship is more evident for women with minimum economic level, which may be due to the fact that the entrepreneurial needs of economically disadvantaged women may be sensitive to changes in surrounding economic conditions [73].

The interaction of life-cycle stage and human capital resolves the paradox in Table 7. For example, women in “poorer” health (Panel E) show the strongest IMIS response because insurance restores their ability to engage in entrepreneurship-a human capital rebound effect. Similarly, the poorest women (Panel F) benefit the most from IMIS because insurance replaces the missing safety net, enabling them to take risks that human capital constraints would prevent them from taking. These patterns highlight the fact that IMIS does not operate in a vacuum, but interacts dynamically with women’s life-cycle orientation and skill pool.

## 5. Discussion

Entrepreneurship research highlights its inherently gendered nature [52,53,69]. Since the mid-1970s, studies on women’s labor in China have consistently emphasized their critical role in the labor market, particularly in entrepreneurship (self-employment), often described as “holding up half the sky.” Although some literature has reported mixed results regarding the relationship between health insurance and women’s entrepreneurship, there is a lack of causal analysis and plausible mechanisms in the Chinese context. This study contributes to filling this gap by identifying an unexpected consequence in China: IMIS encourages women to engage in higher economic risk behaviors, such as self-employment, a form of entrepreneurship. To establish causality, we utilize IMIS as a natural experiment, employing DID model with robustness tests, which confirm IMIS’s empowering effect of IMIS on women’s entrepreneurship.

This institutional breakthrough particularly disrupts China’s legacy Hukou system that traditionally tied welfare access to geographic registration. By enabling rural-urban migrants to maintain continuous coverage – a group showing the strongest entrepreneurial response – IMIS exemplifies policy feedback creating new political constituencies. The system’s portable benefits empower marginalized groups to pursue non-traditional economic paths.

Our study provides evidence that health insurance significantly impacts women’s entrepreneurship. The results indicate that IMIS stimulates women’s entrepreneurship, particularly among younger, better-educated, rural-urban migrants with children, poorer health, and lowest economic level. Life cycle theory and human capital theory can effectively explain these population heterogeneity results [56,67]. The former argues that young people have more resourceful choices about risk attitudes, occupational preferences, and rural-urban mobility, while the second highlights the educational and health strengths of young people, which may motivate them to take advantage of entrepreneurial opportunities. We examine medical expenditure risk, labor mobility, and women’s household bargaining power as key mechanisms. IMIS offers an opportunity to test these mechanisms. First, following IMIS, women can obtain higher reimbursement rates for medical care across regions (provinces and cities), reducing the entrepreneurial risk associated with illness, a factor that tends to threaten women more than men. Additionally, women in the labor force tend to value health insurance more than men [6]. As IMIS narrows the gap between different groups and lowers the burden of subsistence and entrepreneurial challenges, more women may choose to start their own ventures. Second, IMIS subsidizes two-thirds of health insurance premiums for those not employed in the official sector, allowing women to exit the “entrepreneurial lock” of formal employment by providing better health benefits even if they do not hold a formal job. Furthermore, this shift may fuel women’s labor supply and wage earnings, strengthen their bargaining power within the household [55,70], and ultimately provide an incentive for women to leverage family resources when pursuing entrepreneurship. Intra-household dynamics are also an essential mediator of women’s business management capabilities [68,69]. Thus, another possible mechanism is the bargaining power that women have in crucial family matters.

This study provides evidence that IMIS brings additional labor market benefits, particularly in entrepreneurship, which also impacts women. If health insurance programs aimed at enhancing women’s well-being are further expanded, women could potentially make more autonomous labor supply decisions and engage in entrepreneurial initiatives without being constrained by the need for health insurance. This could significantly enhance the efficiency of China’s labor market. This study seeks to contribute to the investigation of the impact of public insurance on entrepreneurship. The results of this study are expected to inform public of the discourse on health insurance and entrepreneurship by providing nuanced insights into the ongoing impact of health care costs on the uninsured and entrepreneurs, particularly during the COVID-19 pandemic. As health insurance coverage improves, there may be an increase in independent female entrepreneurship.

Given the characteristics of the CFPS data, the results of this study are expected to be applicable to all Chinese women. The CFPS is recognized as providing representative data on the financial behavior of the Chinese population. This study included only respondents aged 18–60 without urban employee health insurance, making the results applicable specifically to Chinese adult women under the age of 60. The goal of this study is to ensure that these findings can be replicated in other Chinese samples.

This study emphasizes the significance of China’s unique insurance system, which requires careful consideration when interpreting its applicability in other countries. Given the complexity and heterogeneity of factors that entrepreneurs consider when choosing among multiple health insurance options, it is essential to understand the contextual differences. Additionally, basic health insurance may involve multiple programs, as seen in China and Vietnam, making it necessary to further investigate the applicability of our findings to other countries. The findings, along with research on the “entrepreneurial lock” effect in the US [5], suggest that a basic health insurance system may promote women’s entrepreneurship. However, the generalizability of these findings may be limited due to the differing national entrepreneurial economic environments.

Additionally, this study has several inherent limitations. First, due to data limitations, only four surveys could be analyzed, restricting the ability to comprehensively capture long-term trends. Nonetheless, the findings still provide valuable insights and implications. Furthermore, while this paper provides empirical evidence on the role of IMIS in motivating women’s entrepreneurship, the final outcomes of these entrepreneurial efforts remain uncertain and require further exploration in future research. Second, although the models in this study account for several meaningful controls related to health insurance, data limitations prevented the inclusion of controls for entrepreneurial characteristics. It limits replication to states that lack similar financial resources or complementary reforms (e.g., loosening of household registration), given that the effectiveness of IMIS depends on the financial capacity of states to subsidize premiums. Due to data limitations, our study focuses on entrepreneurial outcomes; however, the policy’s indirect effects on women’s labor supply decisions, financial risk tolerance, and family dynamics merit further study.

Future research deserves to be further developed to help advance the literature on female entrepreneurship. First, building on our findings, future research can utilize fine-grained datasets such as the China Household Finance Survey (CHHS) to refine indicators of entrepreneurship and explore the dynamics of women’s entrepreneurial trajectories to analyze the entry and sustainability of entrepreneurship. Longitudinal studies are needed to track dynamic outcomes, including venture profitability, exit patterns, and human capital reinvestment effects. Second, economic path analysis can unfold the role of IMIS in reducing vulnerability to business closures, increasing career flexibility and lifetime earnings, and the health multipliers generated by portability, which in turn improves maternal and child health outcomes through the provision of uninterrupted care for mobile populations. While our findings highlight IMIS’s potential to disrupt cycles of medical poverty, the long-term effects on intergenerational mobility and gender norm evolution remain untested. Future research should track cohorts over decades to assess whether early-stage entrepreneurship translates into wealth consolidation or systemic norm shifts, particularly in regions with entrenched patriarchal structures.

## 6. Conclusion

This study reveals that China’s Integrated Medical Insurance System (IMIS) has significantly increased women’s willingness to engage in entrepreneurship, with particularly pronounced effects on young women, highly educated women, rural-to-urban migrant women, mothers, and low-income women. By employing robust methods and national-level data from the China Family Panel Study (CFPS), we found that IMIS increased the likelihood of women engaging in entrepreneurship by 9.6 percentage points. This effect primarily stems from IMIS reducing the financial risks associated with medical expenses, enabling women to more easily engage in high-risk occupations such as entrepreneurship, and enhancing their influence in household decision-making. These findings suggest that expanded universal health coverage is not only a tool for social protection but also actively promotes women’s economic participation and empowerment [53,69].

These findings hold important policy implications. Expanding IMIS to cover mental health and preventive care can help address key barriers faced by women entrepreneurs. Combining insurance registration with business support services can reduce financial barriers, such as using IMIS participation as a credit reference for loans. Integrating basic business training into the IMIS platform can efficiently improve entrepreneurial skills. Developing special provisions for mothers and unmarried women can ensure that these programs benefit all women equally. Regular monitoring of outcomes for different groups will help to continuously improve policies.

These research findings are particularly valuable because they demonstrate how health policy can have broad economic impacts beyond the realm of healthcare. By reducing health-related uncertainty and financial constraints, IMIS empowers women to pursue entrepreneurial opportunities they would otherwise avoid [74,75]. This creates a virtuous cycle where improved health security fosters economic activity that in turn can enhance household welfare. For policymakers, this suggests that health policy should be treated not only as social welfare spending, but also as an investment in women’s economic potential and broader development goals. IMIS’s successful experience in promoting women’s entrepreneurship provides valuable insights for other countries seeking to achieve health equity and inclusive economic growth through comprehensive policy measures.

## Supporting information

S1 Appendix Table A1 Definitions of variables. Note: This table shows the summary statistics for the variables in the sample. These social insurance programs include the Urban Employees’ Basic Medical Insurance (UEBMI), the Urban Residents’ Basic Medical Insurance (URBMI), the New Rural Cooperative Medical Insurance System (NRCMS), the Urban Residents’ Basic Medical Insurance URBMI, and the Free Medical Care (FMC). Table A2 Estimation of the placebo effects. Note: (1) The two-sided p-value is the frequency that the absolute values of the placebo effects are greater than or equal to the absolute value of estimated treatment effect; (2) The left-sided (right-sided) p-value is the frequency that the placebo effects are smaller (greater) than or equal to the estimated treatment effect. Table A3 Bacon Decomposition of the TWFE Coefficients. Note: Early_v_Late indicates that early treated as the treatment group and later treated as the control group. Late_v_Early indicates that late treated as the treatment group and early treated as the control group, i.e., a forbidden comparison. Never_v_timing means never treated as a treatment group and timing as a control group. ***, denote significance levels of 1%. Figure B1 Different PSM methods for kernel density plots.
(DOCX)
