# Supplementary material for: Empowering women entrepreneurs: The impact of integrated medical insurance system in China
Source: PLoS One. 2026 Feb 25;21(2):e0337827. doi: 10.1371/journal.pone.0337827 (PMC12935272; doi:10.1371/journal.pone.0337827)
Supplement: S1 Appendix — Table A1 Definitions of variables. Note: This table shows the summary statistics for the variables in the sample. These social insurance programs include the Urban Employees’ Basic Medical Insurance (UEBMI), the Urban Residents’ Basic Medical Insurance (URBMI), the New Rural Cooperative Medical Insurance System (NRCMS), the Urban Residents’ Basic Medical Insurance URBMI, and the Free Medical Care (FMC). Table A2 Estimation of the placebo effects. Note: (1) The two-sided p-value is the frequency that the absolute values of the placebo effects are greater than or equal to the absolute value of estimated treatment effect; (2) The left-sided (right-sided) p-value is the frequency that the placebo effects are smaller (greater) than or equal to the estimated treatment effect. Table A3 Bacon Decomposition of the TWFE Coefficients. Note: Early_v_Late indicates that early treated as the treatment group and later treated as the control group. Late_v_Early indicates that late treated as the treatment group and early treated as the control group, i.e., a forbidden comparison. Never_v_timing means never treated as a treatment group and timing as a control group. ***, denote significance levels of 1%. Figure B1 Different PSM methods for kernel density plots. (DOCX) [file pone.0337827.s001.docx]

**Appendix A Tables**

**Table A1 Definitions of variables.**

| **Variable** | **Definitions** | **Data Source** |
| --- | --- | --- |
| Women’s entrepreneurship | A dummy variable that equals one if woman have engaged in self-employed or started private enterprises in the past year, and 0 otherwise. | China Family Panel Studies (CFPS) |
| IMIS | A dummy variable that equals 1 if the region is covered by IMIS reform, and 0 otherwise. | Manually collected |
| age | Age is defined as the difference between an individual’s survey year and birth year. | CFPS |
| age2 | Age squared/100. | CFPS |
| education | Years of education. | CFPS |
| hukou | Dummy variable that equals 1 if woman is non-farm and 0 otherwise. | CFPS |
| communist | A dummy variable that equals 1 if woman is a member of communist party, and 0 otherwise. | CFPS |
| medsure_dum | Dummy variable that takes 1 if woman has participated insurance and 0 otherwise^^[[1]](#footnote-1)^^. | CFPS |
| health | Self-reported health status based on response to the question “How would you rate your current health status compared to your peers?” a dummy variable equal to 0 if they reported “poor” or “very poor” and 1 otherwise. | CFPS |
| internet | Dummy variable that takes 1 if woman has internet access and 0 otherwise. | CFPS |
| elder_p | Ratio of elderly aged 65 and over to working age population | CFPS |
| child_p | Ratio of children aged 0-14 years to working age population | CFPS |
| familysize | The number of family members | CFPS |
| hhexp | Annual household consumer expenditures | CFPS |
| hhpca | Annual per capita household assets | CFPS |
| clan | Based on the CFPS question “whether a community has clan temples and the number of clan temples”, the number of clan temples in each region  is calculated | CFPS |
| gdp_pc | Per capita gross domestic product in province | China Statistical Yearbook |
| PD | The populations/ per squared kilometer in province | China Statistical Yearbook |
| perhos | No. of hospitals per thousand population in province | China Statistical Yearbook |
| perdocs | No. of doctors per thousand population in province | China Statistical Yearbook |
| unemp | Provincial unemployment rate | China Statistical Yearbook |
| cpi | Provincial consumer price indices | China Statistical Yearbook |

Note: This table shows the summary statistics for the variables in the sample.

**Table A2 Estimation of the placebo effects**

|  | Coefficient | Two-sided | Left-sided | Right-sided |
| --- | --- | --- | --- | --- |
| IMIS | 0.093 | 0.000 | 1.000 | 0.000 |

Note: (1) The two-sided p-value is the frequency that the absolute values of the placebo effects are greater than or equal to the absolute value of estimated treatment effect; (2) The left-sided (right-sided) p-value is the frequency that the placebo effects are smaller (greater) than or equal to the estimated treatment effect.

**Table A3 Bacon Decomposition of the TWFE Coefficients.**

|  | **Beta** | **Total Weight** |
| --- | --- | --- |
| Early_v_Late | 0.0168 | 0.3667 |
| Late_v_Early | 0.0008 | 0.1833 |
| Never_v_timing | 0.0952 | 0.4500 |
| Overall IMIS estimator | 0. 0492^***^ | 1 |

Note: Early_v_Late indicates that early treated as the treatment group and later treated as the control group. Late_v_Early indicates that late treated as the treatment group and early treated as the control group, i.e., a forbidden comparison. Never_v_timing means never treated as a treatment group and timing as a control group. ^***^, denote significance levels of 1%.

**Appendix B Figures**

Figure B1 reports the kernel density plots of the treatment and control groups before and after matching under the methods of nearest neighbors matching, kernel matching, and radius caliper matching. It can be seen that the deviation of the two kernel density curves before and after matching is relatively large regardless of the method, but the distance between the mean lines is shortened and the two curves are closer after matching, so it can be explained to some extent that the PSM has the effect of reducing the bias of sample selectivity.


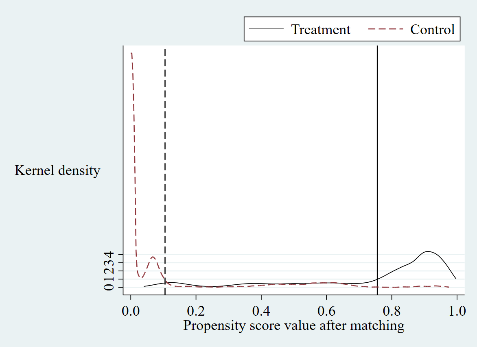

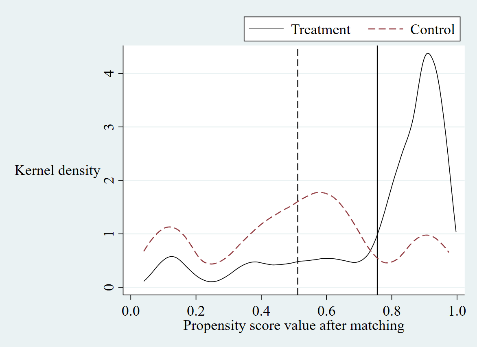


Panel A Nearest neighbors matching


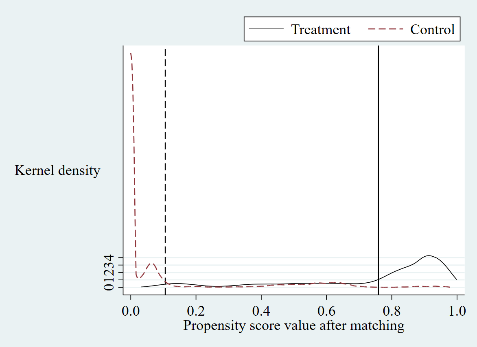

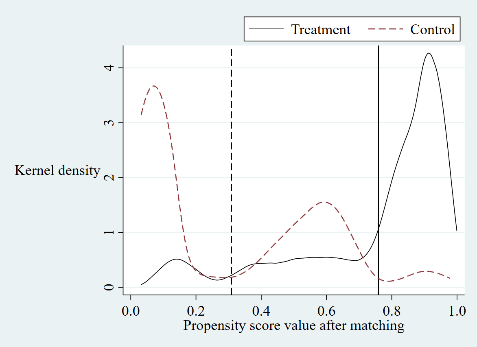


Panel B Kernel matching


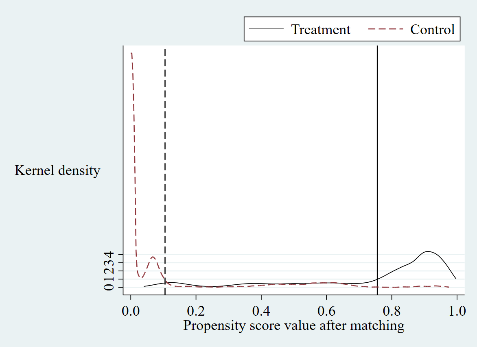

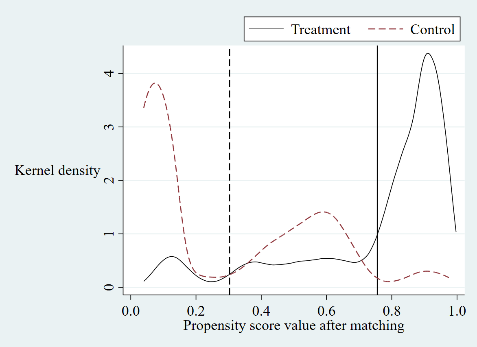


Panel C Radius caliper matching

**Figure B1 Different PSM methods for** **kernel density plots.**

1. These social insurance programs include the Urban Employees’ Basic Medical Insurance (UEBMI), the Urban Residents’ Basic Medical Insurance (URBMI), the New Rural Cooperative Medical Insurance System (NRCMS), the Urban Residents’ Basic Medical Insurance URBMI, and the Free Medical Care (FMC). [↑](#footnote-ref-1)
